# Socioeconomic pattern of breastfeeding in sub-Saharan Africa: an individual participant data meta-analysis of six longitudinal cohorts

**DOI:** 10.1136/bmjph-2024-001298

**Published:** 2025-03-18

**Authors:** Shamsudeen Mohammed, Clara Calvert, Emily L Webb, Judith R Glynn, Suzanne Filteau, Alison Price, Albert Dube, Joseph O Mugisha, Ronald Makanga, Milly Marston, Laura Oakley

**Affiliations:** 1 Department of Non-communicable Disease Epidemiology, London School of Hygiene & Tropical Medicine, London, UK; 2 Usher Institute, The University of Edinburgh , Edinburgh, UK; 3 Department of Infectious Disease Epidemiology, London School of Hygiene & Tropical Medicine, London, UK; 4 Department of Population Health, London School of Hygiene & Tropical Medicine , London, UK; 5 Malawi Epidemiology and Intervention Research Unit, Lilongwe, Malawi; 6 MRC/UVRI and LSHTM Uganda Research Unit, Entebbe, Wakiso, Uganda; 7 Centre for Fertility and Health, Norwegian Institute of Public Health, Oslo, Norway

**Keywords:** Epidemiology, Public Health, Community Health, Sociodemographic Factors

## Abstract

**Background:**

Breastfeeding rates in sub-Saharan Africa (SSA) are declining, and at the current rate, only four African countries will meet the WHO’s 2030 exclusive breastfeeding target. We examined the association between maternal socioeconomic status (SES) and breastfeeding practices in SSA.

**Methods:**

Six cohorts in Ethiopia, Malawi, Uganda and Zambia, with 11 863 participants, were analysed. Data for the cohorts were collected between 2000 and 2021, covering births from 2000 to 2019. SES exposures were maternal education and household income. Breastfeeding outcomes included ever breastfed, early initiation of breastfeeding (Ethiopia only), exclusive breastfeeding for ≥4 months or ≥6 months, and continued breastfeeding for ≥1 year. Risk ratios from multivariable Poisson regression models for individual cohorts were pooled in a random-effects meta-analysis to assess the effects of SES on breastfeeding, adjusting for confounders.

**Results:**

Meta-analysis found no evidence of a difference in ever breastfeeding between mothers with secondary or tertiary education and those with primary/no education. Mothers with secondary education (adjusted risk ratio (aRR)=1.11, 95% CI=1.01 to 1.21) and those from middle-wealth households (aRR=1.12, 95% CI=1.01 to 1.24) were more likely to initiate breastfeeding early than those with primary/no education or low household wealth, but there was no evidence of association in the tertiary education and higher-wealth groups. The association between maternal education and exclusive breastfeeding for ≥4 months and ≥6 months varied across cohorts, with no evidence of association in most cohorts. Overall, household wealth was not associated with exclusive breastfeeding for ≥4 months or ≥6 months. The meta-analysis showed no evidence of association between household wealth and breastfeeding for ≥1 year, but mothers with tertiary education were less likely (aRR=0.93, 95% CI=0.88 to 0.99) to breastfeed for ≥1 year than those with primary or no education.

**Conclusion:**

We observed no clear socioeconomic pattern in breastfeeding, contrasting with patterns observed in high-income countries.

**WHAT IS ALREADY KNOWN ON THIS TOPIC**

- In high-income countries, evidence consistently indicates that women of higher socioeconomic status exclusively breastfeed for a longer duration than those of lower socioeconomic status.
- Recent analyses in sub-Saharan Africa reveal mixed findings on the association between socioeconomic status and breastfeeding practices, leaving uncertainty about how socioeconomic factors impact breastfeeding in the region.

**WHAT THIS STUDY ADDS**

- Two-thirds of mothers initiated breastfeeding within an hour of birth, and 40.2% to 67.9% exclusively breastfed for 6 months, with most mothers breastfeeding for at least 1 year.
- In contrast to findings from high-income countries, there was no clear socioeconomic pattern in ever breastfeeding, breastfeeding initiation, exclusive breastfeeding and duration of any breastfeeding.

**HOW THIS STUDY MIGHT AFFECT RESEARCH, PRACTICE OR POLICY**

- Researchers investigating the effects of breastfeeding on outcomes such as childhood illnesses, cognition and nutrition in sub-Saharan Africa should consider the implications of our findings for their research and modelling strategies.

## Background

Sub-Saharan Africa (SSA) continues to face substantial challenges in infant and young child nutrition, including a continued decline in breastfeeding rates among children under 2 years between 2000 and 2019,^1^ despite the progress in feeding practices since the adoption of the Innocenti Declaration in 1990 to reverse declining breastfeeding rates.^2^ At the current rate of decline, projections indicate that only four African countries will meet the WHO’s target of achieving a 70% prevalence of exclusive breastfeeding in the first 6 months after birth by 2030.^3^ Increased exclusive breastfeeding rates to meet the WHO 2030 targets^4^ could prevent nearly 200 000 under-5 deaths in low and middle-income countries (LMICs) between 2020 and 2030.^5^

Breastfeeding in SSA is influenced by multiple factors, including social and cultural norms, access to healthcare, maternal and infant characteristics, and community and family support for breastfeeding.^6^,^11^ Socioeconomic factors such as income, education and occupation, which influence access to resources, healthcare services and educational resource utilisation, could also impact breastfeeding. In high-income countries, there is strong, consistent evidence that women with higher education and income are more likely to breastfeed, and for a longer duration, than women with lower education and income.^12^,^15^ However, it remains unclear if there is an association between socioeconomic status and breastfeeding in LMIC settings.

Recent analyses show mixed findings on the association between socioeconomic status and breastfeeding practices in LMICs. In two analyses of cross-sectional data from the 2010 to 2018 Demographic and Health Surveys (DHS) for over 85 LMICs, children from the poorest families had higher exclusive and continued breastfeeding rates than those from the richest families.^16 17^ However, a study of 2015 to 2019 DHS data from 16 SSA countries found no differences in exclusive breastfeeding by maternal education or income status.^18^ While that study also found no differences in breastfeeding initiation by maternal education or income,^18^ another study using DHS data for 32 SSA countries from 2010 to 2020 found that higher education and income levels were associated with higher odds of breastfeeding initiation.^8^

A better understanding of socioeconomic patterns in breastfeeding can guide policies and interventions appropriate to women from various backgrounds. It can also help researchers examine the relationship between breastfeeding and outcomes in education, cognitive development and childhood health by showing how various socioeconomic conditions shape breastfeeding behaviour. Previous studies have been from cross-sectional surveys, with notable limitations^19 20^ and inconsistent findings. For instance, the DHS often collects data on exclusive breastfeeding by assessing the current status of mothers using the 24-hour recall method. This method has been found to overestimate breastfeeding rates and may lead to misleading conclusions when compared with the ‘exclusive breastfeeding since birth’ approach, which is commonly employed to collect breastfeeding data in prospective studies.^20 21^

Therefore, in this study, we investigated the socioeconomic patterns of breastfeeding in SSA using data from six prospective longitudinal birth cohorts. Unlike cross-sectional surveys, the prospective design of these birth cohorts allowed data collection at several time points, thereby reducing recall bias and providing a more accurate and temporally aligned assessment of breastfeeding practices relative to socioeconomic exposures. Additionally, by bringing together data from six distinct cohorts, our study draws on a range of settings within SSA, improving our ability to consider how various cultural practices, data collection methods and temporal factors may influence the association between socioeconomic status and breastfeeding.

## Methods

Data for this analysis came from six prospective, longitudinal cohort studies conducted in four SSA countries: the Performance Monitoring for Action Ethiopia Cohort 1 (PMA-Cohort1-Ethiopia)^22^; the Performance Monitoring and Accountability 2020 Maternal and Newborn Health Survey in Ethiopia (PMA-MNH-Ethiopia)^23^; the Karonga Health and Demographic Surveillance System site in Malawi (Karonga-HDSS-Malawi)^24^; the General Population Cohort from the Kyamulibwa Health and Demographic Surveillance System in rural Southwestern Uganda (GPC-Uganda)^25^; the Chilenje Infant Growth, Nutrition, and Infection Study in Zambia (CIGNIS-Zambia)^26^; and the Breastfeeding and Postpartum Health study in Zambia (BFPH-Zambia).^27 28^

The Ethiopia cohorts (PMA-Cohort1-Ethiopia and PMA-MNH-Ethiopia) recruited participants using a multistage sampling approach to collect data on various reproductive, maternal and newborn health (RMNH) indicators. Karonga-HDSS-Malawi and GPC-Uganda undertake continuous demographic surveillance in geographically defined populations to collect information on vital events, household members’ characteristics, and various determinants of health. Participants for CIGNIS-Zambia and BFPH-Zambia were recruited from clinics. CIGNIS-Zambia was a randomised, double-blind, controlled trial to assess the impact of two locally made complementary foods on stunting.^26^ BFPH-Zambia was a prospective cohort study to investigate the risk for subclinical mastitis, breast milk HIV viral load, and postpartum morbidity among lactating women.^27 28^ Children in the cohorts were born in 2000 or later. A description of each cohort is presented in online supplemental table 1, with detailed descriptions published elsewhere.^23^,^2628^

## Breastfeeding

Breastfeeding information was collected at multiple time points in each cohort. Online supplemental table 1 shows the specific breastfeeding-related information collected in each cohort. In general, mothers were asked about breastfeeding practices in the 24 hours preceding the interview at each round or visit, including whether the child was ever breastfed, when the mother began breastfeeding (in hours) after delivery, the child’s current breastfeeding status and the child’s age when breastfeeding stopped. Mothers were also asked about the number of months the child was fed only breastmilk before other foods and liquids were introduced. Not all these indicators were available for all cohorts. Table 1 presents the breastfeeding indicators available for each cohort analysed.

**Table 1:** Definitions of breastfeeding indicators

Breastfeeding indicator	Definition	Groups compared	Cohorts
Ever breastfed	Proportion of children who were ever fed breastmilk.	Ever breastfed vs never breastfed	PMA-Cohort1-Ethiopia PMA-MNH-Ethiopia Karonga-HDSS-Malawi GPC-Uganda CIGNIS-Zambia BFPH-Zambia
Early initiation of breastfeeding	Proportion of children who were put to the breast within 1 hour of birth.	Initiate breastfeeding ≤1 hour after birth vs >1 hour after birth	PMA-Cohort1-Ethiopia PMA-MNH-Ethiopia
Exclusive breastfeeding for ≥4 months	Proportion of infants who were fed only breastmilk and no other liquids or solids except for oral rehydration salt, drops and syrups (vitamins, mineral supplements or medicines) for at least 4 months after birth.	Exclusive breastfeeding for ≥4 months vs introduced other liquids or solids for feeding before 4 months	PMA-Cohort1-Ethiopia Karonga-HDSS-Malawi GPC-Uganda BFPH-Zambia
Exclusive breastfeeding for ≥6 months	Proportion of infants who were fed only breastmilk and no other liquids or solids except for oral rehydration salt, drops and syrups (vitamins, mineral supplements or medicines) for at least 6 months after birth.	Exclusive breastfeeding for ≥6 months vs introduced other liquids or solids for feeding before 6 months	PMA-Cohort1-Ethiopia Karonga-HDSS-Malawi GPC-Uganda
Continued breastfeeding for ≥1 year	Proportion of children who were fed breastmilk for at least 1 year after birth.	Continued breastfeeding for ≥1 year vs did not breastfeed at 1 year or beyond	PMA-Cohort1-Ethiopia Karonga-HDSS-Malawi GPC-Uganda CIGNIS-Zambia

### Measures of socioeconomic status

The measures of socioeconomic status were maternal education and asset-based household wealth. Data for these measures were collected around the time of the child’s birth. Information on maternal education was from the mothers’ responses to the question on their highest level of education. In general, household wealth was estimated in each cohort based on principal component analysis of a range of household assets, dwelling building materials, access to utilities, and livestock ownership, depending on what was available. Each household was assigned a score, which was then divided into quantiles based on the distribution of the score from lowest to highest household wealth. Detailed descriptions of the items included in the calculation of each cohort’s wealth index are published elsewhere.^2331^,^34^ While maternal employment may influence breastfeeding practices, reliable and consistent employment data were not available across all six cohorts. To ensure comparability, we focused on maternal education and household wealth, as these were consistently measured and are widely recognised as core indicators of socioeconomic status in SSA.

## Data analysis

The prevalence of the breastfeeding indicators available for each cohort was calculated and summarised across participant characteristics. We classified maternal education into three categories (data from Karonga-HDSS-Malawi, CIGNIS-Zambia and BFPH-Zambia were originally grouped in these categories): none/primary, secondary and tertiary. Similarly, household wealth was classified into three categories: low, middle and high.

A two-stage individual participants data (IPD) meta-analysis^35^ was used to determine the association between measures of socioeconomic status and breastfeeding outcomes, with low socioeconomic status (none/primary education or low household wealth) as the reference group.

In the first stage, univariable and multivariable Poisson regression models with robust error variance^36^,^38^ were constructed separately for each cohort and used to estimate risk ratios with corresponding 95% CIs. The multivariable models were performed as a complete case analysis adjusting for potential confounders available for each cohort, including maternal age, maternal HIV status, marital status, place of residence, and parity or birth order. Analysis for the indicators ‘ever breastfed’ for the Karonga HDSS, GPC-Uganda, PMA-MNH-Ethiopia and BFPH-Zambia and ‘continued breastfeeding’ for Karonga HDSS were excluded from the regression analysis because almost every mother in these cohorts breastfed or continued breastfeeding for at least 1 year. In the regression models for the two Ethiopian cohorts, we used Stata’s survey commands to account for the multistage sampling design.

In the second stage, the study-specific adjusted risk ratios were pooled in a random-effects meta-analysis to give overall estimates of the association of each maternal educational level and household wealth level with breastfeeding. Forest plots were used to display the meta-analysis results. Between-cohort variance was assessed using the tau-squared (τ^2^) test,^39 40^ and the percentage of variability in the cohort-specific estimates attributable to true heterogeneity, not sampling error, was evaluated with Higgins and Thompson’s I^2^ statistic.^41^ Given that some heterogeneity is expected when combining estimates across different studies, I^2^ values of 25%, 50% and 75% were considered low, moderate and high heterogeneity, respectively.^42 43^ The p value from Cochran’s Q statistic test was further used to assess for statistical evidence of heterogeneity.^40^ Because the number of studies in a meta-analysis and the precision of estimates can influence these heterogeneity measures,^4041 44^,^46^ we also visually inspected the direction and magnitude of effect estimates to determine heterogeneity across the cohorts. All pooled estimates are presented in forest plots, but pooled estimates are only interpreted where there was no strong evidence of heterogeneity. Random-effects multivariable meta-regression of study-level covariates was performed to investigate sources of between-study heterogeneity unless data were sparse or strongly correlated.

Considering the extensive breastfeeding education efforts targeting mothers with HIV and the fact that breastfeeding recommendations differ for mothers living with HIV, particularly during the periods most of the cohorts collected data, sensitivity analysis was conducted to evaluate the robustness of the results to any potential influence from this group. In the sensitivity analysis, we repeated all the analyses excluding mothers known to be living with HIV for the four cohorts with HIV data available (Karonga-HDSS-Malawi, GPC-Uganda, BFPH-Zambia and CIGNIS-Zambia).

### Patient and public involvement

Patients or the public were not involved in the design, or conduct, or reporting, or dissemination plans of our research.

## Results

The study sample consisted of 11 863 participants from six cohorts across four SSA countries (Ethiopia, Malawi, Uganda and Zambia), with sample sizes ranging from 315 in the PMA-MNH-Ethiopia cohort to 6861 in the GPC-Uganda cohort (table 2). The majority of mothers were aged 20–29, married, had primary education or less, lived in low-wealth households and were known to be HIV-negative (for the four cohorts with HIV data available) (table 3 and online supplemental table 2).

**Table 2:** Prevalence of breastfeeding in six sub-Saharan African birth cohorts

Country	Ethiopia	Ethiopia	Malawi	Uganda	Zambia	Zambia
Cohort acronym	PMA-Cohort1-Ethiopia	PMA-MNH-Ethiopia	Karonga-HDSS-Malawi	GPC-Uganda	CIGNIS-Zambia	BFPH-Zambia
Dates of data collection*	2019–2021	2016–2017	2002–2005	2000–2011	2005–2009	2001–2003
Year of birth	2019	2016	2002–2004	2000–2011	2005	2001
Sample size	2038	315	1464	6861	811	374
	n (%)	n (%)	n (%)	n (%)	n (%)	n (%)
Ever breastfed						
No	36 (1.8)	0 (0.0)	6 (0.4)	50 (0.7)	51 (6.3)	0 (0.0)
Yes	2002 (98.2)	315 (100.0)	1458 (99.6)	6811 (99.3)	760 (93.7)	374 (100.0)
Early initiation of breastfeeding						
After 1 hour	660 (32.6)	116 (36.9)	–	–	–	–
Within 1 hour	1364 (67.4)	199 (63.1)	–	–	–	–
Exclusive breastfeeding for 4 months						
<4 months	106 (5.8)	–	432 (29.6)	1619 (27.8)	–	246 (65.8)
≥4 months	1711 (94.2)	–	1026 (70.4)	4204 (72.2)	–	128 (34.2)
Exclusive breastfeeding for 6 months						
<6 months	584 (32.1)	–	872 (59.8)	2571 (44.2)	–	–
≥6 months	1234 (67.9)	–	586 (40.2)	3252 (55.8)	–	–
Continued breastfeeding						
<1 year	52 (2.6)	–	11 (0.8)	1418 (31.7)	263 (34.6)	–
≥1 year	1968 (97.4)	–	1405 (99.2)	3052 (68.3)	497 (65.4)	–

* The period during which the data analysed in this study were collected.

**Table 3:** Distribution of the breastfeeding indicators by maternal socioeconomic status for each cohort

	Total	Ever breastfed	Breastfeeding initiation within 1 hour after birth	Exclusive breastfeeding ≥4 months	Exclusive breastfeeding ≥6 months	Continued breastfeeding ≥1 year
**PMA-Cohort1-Ethiopia** *						
Maternal education						
None/primary	1708 (81.5)	1632 (98.1)	1103 (66.6)	1374 (93.9)	997 (68.2)	1612 (98.3)
Secondary	302 (14.4)	286 (99.0)	210 (73.6)	256 (94.4)	188 (69.4)	281 (94.8)
Higher	86 (4.1)	84 (98.8)	51 (62.4)	81 (97.5)	49 (58.3)	75 (89.2)
Household wealth level						
Low	852 (40.7)	808 (97.2)	537 (64.6)	683 (93.7)	501 (68.8)	799 (98.9)
Middle	420 (20.0)	400 (97.9)	284 (69.1)	331 (93.3)	240 (67.6)	396 (98.7)
High	824 (39.3)	794 (99.5)	544 (69.5)	697 (95.1)	492 (67.1)	773 (95.4)
PMA-MNH-Ethiopia*						
Maternal education						
None/primary	289 (89.2)	–	176 (62.7)	–	–	–
Secondary	32 (9.7)	–	20 (64.7)	–	–	–
Higher	4 (1.1)	–	3 (76.4)	–	–	–
Household wealth level						
Low	123 (37.4)	–	69 (58.0)	–	–	–
Middle	110 (33.5)	–	74 (71.2)	–	–	–
High	96 (29.1)	–	55 (60.4)	–	–	–
**Karonga-HDSS-Malawi**						
Maternal education						
None/primary	1096 (78.8)	1090 (99.6)	–	744 (68.3)	421 (38.6)	1050 (99.5)
Secondary	295 (21.2)	293 (99.3)	–	222 (75.8)	127 (43.3)	286 (99.0)
Household wealth						
Low	356 (35.1)	353 (99.4)	–	225 (63.7)	121 (34.3)	344 (100.0)
Middle	340 (33.5)	339 (99.7)	–	233 (68.7)	134 (39.5)	323 (99.7)
High	319 (31.4)	317 (99.7)	–	239 (75.4)	132 (41.6)	309 (100.0)
**GPC-Uganda**						
Maternal education						
None/primary	1205 (70.1)	13 (100.0)	–	755 (69.2)	563 (51.6)	725 (85.2)
Secondary	440 (25.6)	427 (100.0)	–	296 (74.4)	227 (57.0)	276 (83.1)
Higher	75 (4.4)	71 (100.0)	–	61 (91.0)	55 (82.1)	38 (77.5)
Household wealth level						
Low	3897 (45.6)	2818 (99.2)	–	1797 (72.9)	1404 (57.0)	1387 (69.8)
Middle	1688 (19.7)	1206 (99.3)	–	754 (72.8)	586 (56.6)	567 (66.9)
High	2966 (34.7)	2046 (99.3)	–	1300 (72.5)	1011 (56.4)	1007 (68.6)
**CIGNIS-Zambia**						
Maternal education						
None/primary	269 (33.2)	253 (94.1)	–	–	–	154 (60.9)
Secondary	309 (38.1)	292 (94.5)	–	–	–	198 (67.8)
Higher	233 (28.7)	215 (92.3)	–	–	–	145 (67.4)
Household wealth level						
Low	325 (40.1)	309 (95.1)	–	–	–	200 (64.7)
Middle	162 (20.0)	149 (92.0	–	–	–	95 (63.8)
High	324 (39.9)	302 (93.2)	–	–	–	202 (66.9)
**BFPH-Zambia**						
Maternal education						
None/primary	57 (15.2)	–	–	18 (31.6)	–	–
Secondary	211 (56.4)	–	–	78 (37.0)	–	–
Higher	106 (28.3)	–	–	32 (30.2)	–	–
Household wealth level						
Low	178 (47.6)	–	–	65 (36.5)	–	–
Middle	178 (47.6)	–	–	59 (33.1)	–	–
High	18 (4.8)	–	–	4 (22.2)	–	–

Not all participants in the total sample had information on all the breastfeeding indicators.

* Percentages and counts are weighted estimates.

### Prevalence of Breastfeeding

The prevalence of the breastfeeding indicators in each cohort is shown in table 2. Nearly all the mothers breastfed their babies, with the lowest prevalence (93.7%) in the CIGNIS-Zambia cohort (particularly among mothers living with HIV (online supplemental table 2). Both Ethiopian cohorts with early initiation data had two-thirds of mothers breastfeeding within an hour of birth. Exclusive breastfeeding for at least 4 months was lowest in BFPH-Zambia (34.2%) and highest in PMA-Cohort1-Ethiopia (94.2%). Exclusive breastfeeding for 6 months or longer was 40.2% in Karonga-HDSS-Malawi, 55.8% in GPC-Uganda and 67.9% in PMA-Cohort1-Ethiopia. Most women breastfed for at least a year, with prevalence ranging from 65.4% in CIGNIS-Zambia to 99.2% in Karonga-HDSS-Malawi.

### Association between socioeconomic status indicators and breastfeeding

**Figure 1:**
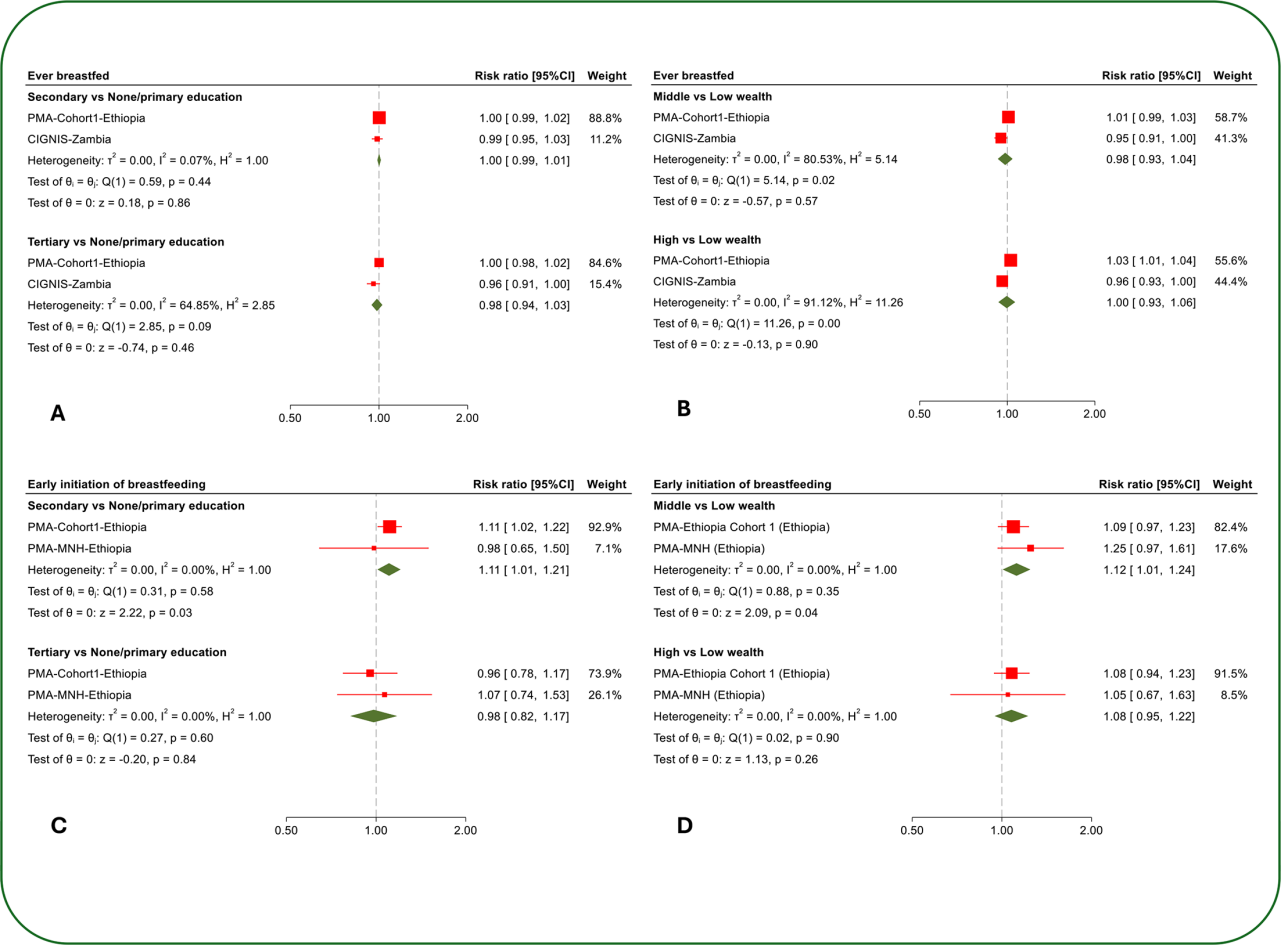
1 The association of maternal education and household wealth with whether a mother ever breastfed (A and B) and breastfeeding initiation within 1 hour after birth (C and D). The cohort-specific estimates are adjusted risk ratios. PMA-Cohort1-Ethiopia: adjusted for child sex, place of residence, maternal age, parity and maternal marital status. CIGNIS-Zambia: adjusted for child sex, child HIV status, maternal HIV status, number of siblings, maternal age and maternal marital status. PMA-MNH-Ethiopia: adjusted for child sex, place of residence, maternal age, parity and maternal marital status.

The distribution of the breastfeeding indicators by maternal socioeconomic status for each cohort is presented in table 3 and online supplemental table 3 presents the unadjusted and adjusted cohort-specific risk ratios for the association of maternal education and household wealth with the breastfeeding indicators. Figures1^2^ show the pooled results from the meta-analysis of the adjusted risk ratios.

**Figure 2:**
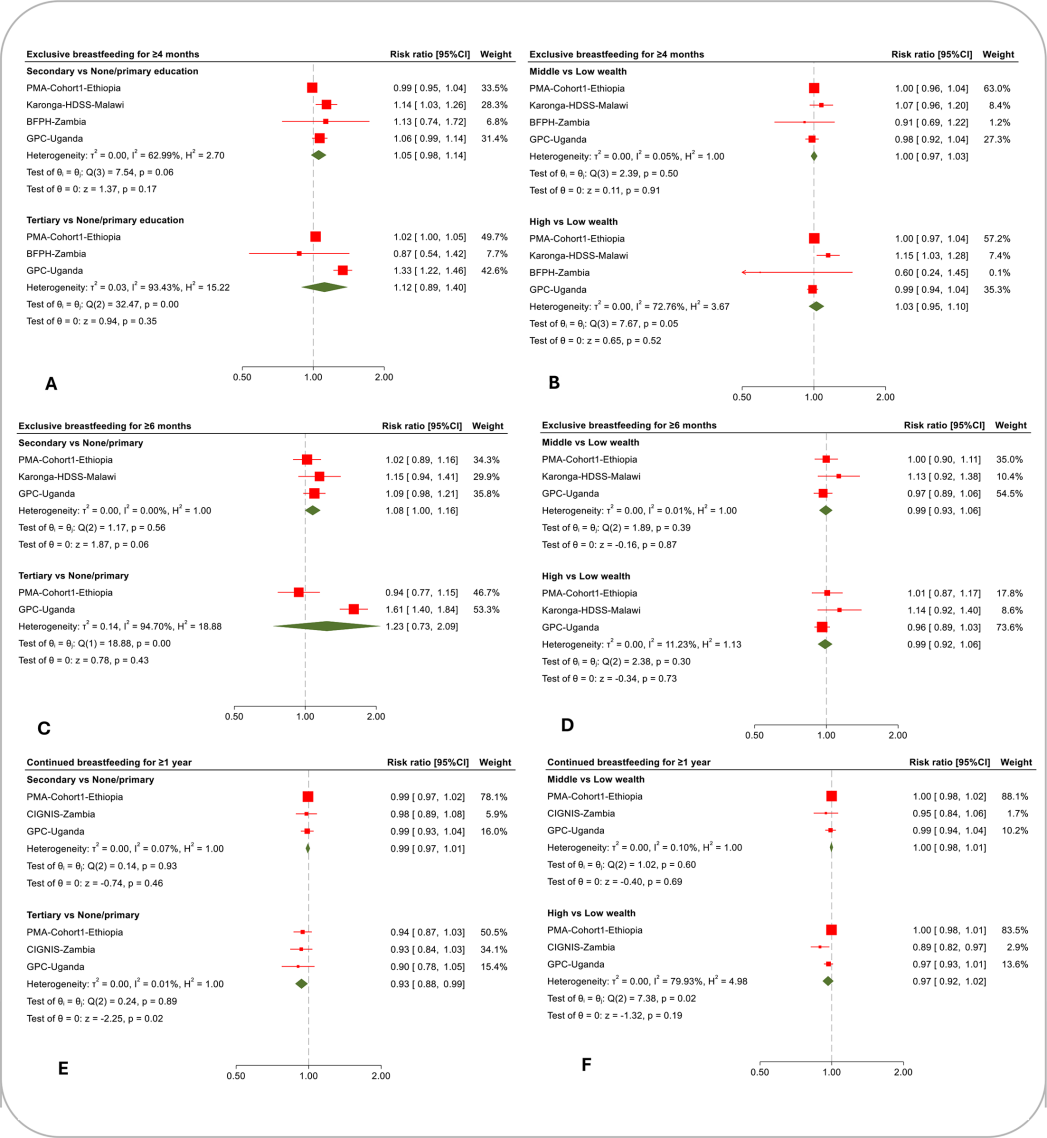
The association of maternal education and household wealth with exclusive breastfeeding for ≥4 months (A and B), exclusive breastfeeding for ≥6 months (C and D) and continued breastfeeding (E and F). The cohort-specific estimates are adjusted risk ratios. PMA-Cohort1-Ethiopia: adjusted for child sex, place of residence, maternal age, parity and maternal marital status. Karonga-HDSS-Malawi: adjusted for child sex, birth order, distance to a tarmac road, maternal HIV status and maternal age. BFPH-Zambia: adjusted for child sex, maternal age, maternal HIV status and maternal marital status. GPC-Uganda: adjusted for child sex, maternal age, maternal HIV status and maternal marital status.

## Ever Breastfed

There was no difference in ever breastfeeding between mothers with secondary (adjusted risk ratio (aRR) 1.00, 95% CI 0.99 to 1.02) or tertiary (aRR 1.00, 95% CI 0.98 to 1.02) education and those with primary or no education in PMA-Cohort1-Ethiopia (online supplemental table 3). Estimates for CIGNIS-Zambia showed no evidence of a difference in ever breastfeeding by maternal educational level. The meta-analysis showed no evidence that breastfeeding differed by maternal educational level (figure 1).

The cohort-specific analysis showed some evidence that mothers from middle (aRR 1.01, 95% CI 0.99 to 1.03) and higher (aRR 1.03, 95% CI 1.01 to 1.04) wealth households were slightly more likely (1%–3% higher) to ever breastfeed compared with mothers from low-wealth households in PMA-Cohort1-Ethiopia (online supplemental table 3). However, in the CGINIS-Zambia cohort, there was some evidence that mothers from middle (aRR 0.95, 95% CI 0.91 to 1.00) and higher (aRR 0.96, 95% CI 0.93 to 1.00) wealth households were less likely to ever breastfeed than mothers from low-wealth households. Heterogeneity in the cohort-specific estimates (figure 1) was high (middle-wealth: I^2^=80.5%, p=0.02; higher-wealth: I^2^=91.1%, p<0.001). Data from the two cohorts were too sparse to investigate the sources of heterogeneity.

### Early initiation of breastfeeding

Mothers with secondary education (aRR 1.11, 95% CI 1.02 to 1.22) were more likely to initiate breastfeeding within 1 hour after birth than those with primary or no education in PMA-Cohort1-Ethiopia (online supplemental table 3). There was no evidence of an association in the much smaller PMA-MNH-Ethiopia (secondary: aRR 0.98, 95% CI 0.65 to 1.50; tertiary: aRR 1.07, 95% CI 0.74 to 1.53). In the meta-analysis (figure 1), there was evidence that mothers with secondary education (aRR 1.11, 95% CI 1.01 to 1.21, p=0.03) were more likely to initiate breastfeeding within 1 hour after birth than those with primary or no education, but there was no evidence of a difference with tertiary education (aRR 0.98, 95% CI 0.82 to 1.17, p=0.84).

The patterns of the estimates in both the PMA-Cohort1-Ethiopia and PMA-MNH-Ethiopia cohorts were consistent with mothers from middle and higher-wealth households being more likely to initiate breastfeeding within 1 hour after birth than mothers from low-wealth households, but there was no clear evidence of an association within individual cohorts. When the estimates were combined in the meta-analysis (figure 1), there was evidence that mothers from middle-wealth households were more likely to initiate breastfeeding within the first hour after birth than mothers from low-wealth households (aRR 1.12, 95% CI 1.01 to 1.24, p=0.04), but there was little evidence of association for the higher-wealth vs low-wealth group comparison (aRR 1.08, 95% CI 0.95 to 1.22, p=0.26).

### Exclusive breastfeeding for at least four months

There was some evidence that mothers with secondary education in Karonga-HDSS-Malawi and GPC-Uganda and those with tertiary education in PMA-Cohort1-Ethiopia and GPC-Uganda were more likely to exclusively breastfeed for at least 4 months than those with primary or no education (online supplemental table 3). In the meta-analysis (figure 2), there was evidence of substantial heterogeneity in the estimates across the cohorts (secondary: I^2^=63.0%, p=0.06; tertiary: I^2^=93.4%, p<0.001). In multivariable meta-regression, the year breastfeeding data were collected accounted for 39.2% of the between-study heterogeneity (online supplemental table 4). Cohorts that collected data in 2019 or later showed a weaker association between maternal education and exclusive breastfeeding for at least 4 months compared with those that collected breastfeeding data prior to 2019.

There was no evidence of a difference in exclusive breastfeeding for at least 4 months between mothers from middle-wealth and those from low-wealth households in both the cohort-specific analysis (online supplemental table 3) and meta-analysis (figure 2). For the higher-wealth group, there was some evidence in Karonga-HDSS-Malawi that mothers from higher-wealth households were more likely to exclusively breastfeed for at least 4 months than those from low-wealth households (aRR 1.15, 95% CI 1.03 to 1.28), but no evidence of an association in the other cohorts. There was high heterogeneity in the higher-wealth vs low-wealth group analysis (I^2^=72.8%, p=0.05). Multivariable meta-regression showed that differences in sample size accounted for 25.9% of the between-study heterogeneity (online supplemental table 4). Cohorts with 1500 or more participants showed a weaker association between household wealth and exclusive breastfeeding for at least 4 months.

### Exclusive breastfeeding for at least six months

In the cohort-specific analysis, there was no clear evidence for a difference in exclusive breastfeeding for at least 6 months between mothers with secondary education and those with primary or no education (online supplemental table 3). In the meta-analysis (figure 2), mothers with secondary education were slightly more likely than those with primary or no education to exclusively breastfeed for 6 months (aRR 1.08, 95% CI 1.00 to 1.16, p=0.06). In GPC-Uganda, mothers with tertiary education were more likely to exclusively breastfeed for at least 6 months than those with primary or no education (aRR 1.61, 95% CI 1.40 to 1.84), but there was no evidence of association in PMA-Cohort1-Ethiopia (online supplemental table 3). The meta-analysis (figure 2) shows high heterogeneity in the tertiary education group (I^2^=94.7%, p<0.001). The data were too sparse to investigate the sources of the heterogeneity.

In the cohort-specific analyses (online supplemental table 3) and meta-analysis (figure 2), there was no evidence for a difference in exclusive breastfeeding for at least 6 months between mothers from middle-wealth (pooled effect aRR 0.99, 95% CI 0.93 to 1.06, p=0.87) or higher-wealth (pooled effect aRR 0.99, 95% CI 0.92 to 1.06, p=0.73) households and those from low-wealth households.

### Continued breastfeeding for one year or longer

In the cohort-specific analyses, there was no clear evidence for a difference in continued breastfeeding for at least 1 year between mothers with secondary or tertiary education and those with primary or no education (online supplemental table 3), although the adjusted estimates for the tertiary education group were consistently below 1 (aRR 0.90 to 0.94). In the meta-analysis (figure 2), there was some evidence that mothers with tertiary education were less likely to continue breastfeeding for at least 1 year compared with those with primary or no education (aRR 0.93, 95% CI 0.88 to 0.99, p=0.02), but there was no evidence for an association in the secondary education group (aRR 0.99, 95% CI 0.97 to 1.01, p=0.46).

The cohort-specific estimates (online supplemental table 3) and meta-analysis (figure 2) showed no evidence that continued breastfeeding for at least 1 year differed between mothers from middle-wealth households and those from low-wealth households. For higher-wealth, there was evidence for an association in CIGNIS-Zambia where mothers from higher-wealth households were less likely to continue breastfeeding for at least 1 year than those from low-wealth households (aRR 0.89, 95% CI 0.82 to 0.97), but no evidence of association in the other cohorts. Heterogeneity was high in the high-wealth group (I^2^=79.9%, p=0.02). Due to data sparsity, meta-regression to explore heterogeneity was not conducted.

### Sensitivity analysis excluding known HIV-positive mothers

The results from the sensitivity analysis excluding known HIV-positive mothers did not differ substantially from the results of the main analysis, except for ever breastfed in the CIGNIS-Zambia cohort where the confidence intervals became narrow, but with only a marginal change (0.01 to 0.04) in the adjusted RRs (online supplemental figures 1,2).

## Discussion

We assessed breastfeeding patterns in six SSA cohorts and examined the association between measures of socioeconomic status (SES) and five breastfeeding indicators. Breastfeeding was nearly universal across all cohorts, with only a small percentage (1%–6%) of children never breastfed. The prevalence of early initiation of breastfeeding, assessed from cohorts in Ethiopia, aligned with the average prevalence for Eastern and Southern Africa (65%) but was higher than the global average (47%) and the average prevalence for West and Central Africa (46%).^47 48^ We found a higher prevalence of exclusive breastfeeding at 4 months than at 6 months, with the largest decline in exclusive breastfeeding rates from 4 to 6 months observed in Malawi. This was probably because the children in the Malawi cohort were born around the time the WHO revised the recommendation for exclusive breastfeeding from 4 to 6 months.^49 50^ Exclusive breastfeeding rates during the first 6 months of life in Ethiopia and Uganda exceeded the WHO’s target of 50% prevalence by 2025,^4^ but rates across all cohorts were below WHO’s more ambitious target of 70% exclusive breastfeeding prevalence by 2030.^4^ Almost all mothers in Malawi and Ethiopia, as well as the majority of mothers in Uganda and Zambia, continued breastfeeding 1 year postpartum. The CIGNIS-Zambia cohort had the lowest continued breastfeeding prevalence of any cohort, probably because mothers in the study received free complementary foods for their infants at 6 months as part of the study, likely discouraging them from continuing to breastfeed.^26^

Overall, there were no clear, consistent socioeconomic patterns in breastfeeding across the SSA cohorts. The sole exception was that, in the meta-analysis, mothers with primary or no education were more likely to breastfeed for a longer duration than those with higher education. In some individual cohorts, middle SES (secondary education or middle-income) or higher SES (higher wealth or tertiary education) had a modest positive or negative effect on certain breastfeeding indicators, but this was inconsistent and mostly only observed in one of the SES categories. Even though the meta-analysis found evidence that mothers of middle SES (secondary education or middle-income) were slightly more likely to initiate breastfeeding early and exclusively breastfeed for 6 months (in the case of those with secondary education) compared with mothers with low SES (primary or no education, or low wealth), it remains unclear whether these associations were truly driven by socioeconomic advantage and not unmeasured confounders (eg, variations in magnitude of family support), given the lack of evidence among women of higher SES. However, this could be attributed to the small number of mothers in the higher education group across the cohorts. The estimates for the CIGNIS-Zambia cohort were sensitive to maternal HIV status, particularly ever breastfeeding, likely because the study was conducted at the time of the AFASS (Acceptable, Feasible, Affordable, Sustainable and Safe) recommendations for HIV-infected mothers, suggesting that HIV-infected women could choose replacement feeding instead of breastfeeding.^51^ We conducted sensitivity analyses for HIV due to established and shifting breastfeeding guidelines for HIV-infected mothers and the availability of relevant data.

Differences in sample size and year of data collection partially explained heterogeneity in cohort estimates for exclusive breastfeeding for at least 4 months. Data sparsity precluded meta-regression to explore between-study heterogeneity for ever breastfed (which was reported in only two studies), exclusive breastfeeding for at least 6 months and continued breastfeeding for at least 1 year. The heterogeneity in estimates for these outcomes may reflect varying cultural norms around infant feeding, the influence of community support groups, variations in breastfeeding advice from local healthcare professionals, differences in data collection timing across cohorts, differences in the covariates adjusted and their completeness in each cohort, as well as cohort-specific sample characteristics. For example, the lower prevalence of exclusive breastfeeding at 4 months in the BFPH-Zambia cohort was probably because data on breastfeeding were collected more frequently than in the other cohorts, minimising the possibility of overestimation in maternal reporting of feeding practices.

Similar to our findings, several studies from sub-Saharan Africa and other LMICs found no evidence for a clear socioeconomic pattern in breastfeeding.^616^,^18^ However, our findings are in contrast to evidence from high-income countries, where there are clear socioeconomic inequalities and patterns in breastfeeding, with higher levels of maternal education and household income associated with a longer duration of any breastfeeding and exclusive breastfeeding.^12^,^15^

In many SSA communities, breastfeeding is a cultural norm, and new mothers face societal expectations to breastfeed,^11 52^ which likely explains why nearly all mothers in this study breastfeed. In addition, the majority of mothers in SSA have access to strong informal breastfeeding support from peers and family,^53^ which helps fill in breastfeeding knowledge gaps for mothers with no formal education. This support system may explain the absence of clear socioeconomic disparities as it promotes equity in breastfeeding by levelling the playing field and eliminating any potential breastfeeding advantage mothers with higher education may have over those with little to no education. Also, in some cultures, infant and young child feeding decisions are largely influenced by grandmothers and mothers-in-law,^11 53^ regardless of the mother’s socioeconomic status. Their preferences, which may include the early introduction of complementary foods,^10^ may potentially diminish any benefits higher socioeconomic status may have on breastfeeding practises, as observed in this study. These breastfeeding-related cultural norms differ across countries and may further explain the heterogeneity in the estimates for the associations across the cohorts analysed.

Furthermore, mothers with higher levels of formal education are more likely to work outside the home^54^ and may struggle to adhere to recommended breastfeeding practices when returning to work after childbirth,^10 53^ resulting in early cessation. It is also common for working mothers to leave their babies with family members, usually grandmothers, who may introduce new foods to the child.^10^ Less educated mothers are more likely to breastfeed for a longer duration, probably because they are more likely to be self-employed or work for family members with more flexibility for childcare. Additionally, earlier studies have shown that breastmilk substitutes are expensive,^55^ making them relatively accessible to mothers with high SES who may be returning to work less than 6 months postpartum, resulting in the early introduction of other types of milk. Also, mothers with higher SES may perceive formula feeding as a status symbol, demonstrating their wealth and ability to provide their infants with expensive baby food, often incorrectly perceived to be more nutritious than breastmilk. Indeed, studies have shown that the use of breastmilk substitutes is more common among mothers with higher SES than those with low SES in LMICs.^16 17^

Breastfeeding education campaigns should prioritise mothers with lower education levels, focusing on the importance of early initiation and exclusive breastfeeding for 6 months. For mothers with tertiary education, who are more likely to be in formal employment and of higher wealth, interventions should aim to reduce barriers to continued breastfeeding by promoting flexible working arrangements, providing workplace breastfeeding support such as private spaces for breastfeeding or expressing milk, and advocating for extended maternity leave. Education on the long-term benefits of breastfeeding and access to community-based peer support networks can help encourage and sustain appropriate breastfeeding practices across all socioeconomic groups. Training for healthcare workers should be expanded to equip them to be able to support breastfeeding promotion for women from diverse backgrounds, including the ability to engage with culturally sensitive educational materials, involve community leaders and stakeholders in promotion activities, and assist mothers in managing breastfeeding challenges at work or home.

Our study has several strengths, including analysis of a large, diverse sample of mothers from multiple SSA countries across different periods, allowing us to capture nuances across regions and study periods. In addition, we assessed multiple breastfeeding indicators, allowing for the assessment of the impact of SES on short-term and long-term breastfeeding outcomes. The use of meta-analysis to pool estimates across the cohorts increased the power of the study to detect a difference. However, heterogeneity for some comparisons precluded the interpretation of pooled estimates. The main limitation of this study is that breastfeeding data was self-reported by mothers, and recall or social desirability biases may have resulted in misclassification of children. For example, in the PMA-Cohort1-Ethiopia, the question about when (minutes, hours or days) mothers initiated breastfeeding after birth was asked 6 weeks after childbirth. Previous studies in Ethiopia have shown that most mothers are unable to recall the exact timing of breastfeeding initiation several weeks after birth, mainly based on a single question of when breastfeeding was initiated.^56 57^ Mothers likely provided responses that they considered socially acceptable rather than their actual practices. This could explain the higher prevalence of breastfeeding initiation in the Ethiopian cohorts. Nevertheless, most cohorts collected breastfeeding data at multiple time points, at regular intervals, in the first 2–3 years postpartum, so recall bias and misclassification are likely minimal. Due to data limitations and sparsity, we could not disentangle all sources of heterogeneity, particularly for ever breastfed (reported in only two studies), exclusive breastfeeding for at least 6 months and continued breastfeeding for at least 1 year. However, some sources of heterogeneity for exclusive breastfeeding for at least 4 months were investigated. It is possible that the unavailability of maternal employment data may mask relations between socioeconomic conditions and breastfeeding practices. Future studies would benefit from examining maternal employment indicators alongside education and wealth. We also encourage future studies to collect data on social support and cultural breastfeeding norms to clarify their role in the relationship between SES and breastfeeding in SSA. Data for this analysis were from four countries, so caution should be exercised in generalising our findings beyond these country settings.

## Conclusion

Despite the high acceptance of breastfeeding in SSA, healthcare providers must continue to promote breastfeeding to sustain the relatively high breastfeeding rates. Further studies to understand the factors influencing breastfeeding in SSA could inform strategies to improve optimal breastfeeding rates. Researchers investigating the impact of breastfeeding on outcomes such as childhood illnesses, cognitive and educational achievements, and nutrition in SSA should carefully consider the implications of these findings for their research, particularly the lack of consistent socioeconomic patterns in breastfeeding.

## Supplementary material

10.1136/bmjph-2024-001298 online supplemental file 1

10.1136/bmjph-2024-001298 online supplemental file 2

10.1136/bmjph-2024-001298 online supplemental file 3

10.1136/bmjph-2024-001298 online supplemental file 4

10.1136/bmjph-2024-001298 online supplemental file 5

## Data Availability

Data may be obtained from a third party and are not publicly available.
