# Supplementary material for: Socioeconomic pattern of breastfeeding in sub-Saharan Africa: an individual participant data meta-analysis of six longitudinal cohorts
Source: BMJ Public Health. 2025 Mar 18;3(1):e001298. doi: 10.1136/bmjph-2024-001298 (PMC12107469; doi:10.1136/bmjph-2024-001298)
Supplement: online supplemental file 1 [file bmjph-3-1-s001.docx]

**(A)**

**(B)**

**Supplementary Figure 1 The association of maternal education and household wealth with whether a mother ever breastfed (A and B) and continued breastfeeding for one year or longer (C and D) among known HIV-negative mothers. The cohort-specific estimates are adjusted risk ratios. PMA-Cohort1-Ethiopia: adjusted for child sex, place of residence, maternal age, parity, and maternal marital status. CIGNIS-Zambia: adjusted for child sex, child HIV status, number of siblings, Maternal age, and maternal marital status. GPC-Uganda: adjusted for child sex, maternal age, maternal HIV status, and maternal marital status.**

**(C)**

**(D)**

**(A)**

**(C)**

**Supplementary Figure 2 The association of maternal education and household wealth with exclusive breastfeeding for ≥4 months (A and B) and exclusive breastfeeding for at least six months (C and D) among known HIV-negative mothers. The cohort-specific estimates are adjusted risk ratios. PMA-Cohort1-Ethiopia: adjusted for child sex, place of residence, maternal age, parity, and maternal marital status. Karonga-HDSS-Malawi: adjusted for child sex, birth order, distance to a tarmac road, maternal HIV status, and maternal age. BFPH-Zambia: adjusted for child sex, maternal age, maternal HIV status, and maternal marital status. GPC-Uganda: adjusted for child sex, maternal age, maternal HIV status, and maternal marital status.**

**(B)**

**(D)**
