# Supplementary material for: Socioeconomic pattern of breastfeeding in sub-Saharan Africa: an individual participant data meta-analysis of six longitudinal cohorts
Source: BMJ Public Health. 2025 Mar 18;3(1):e001298. doi: 10.1136/bmjph-2024-001298 (PMC12107469; doi:10.1136/bmjph-2024-001298)
Supplement: online supplemental file 2 [file bmjph-3-1-s002.docx]

**Supplementary Table 1 Cohort description and breastfeeding measurement**

| **Cohort acronym** | **Country** | **Cohort description** | **Measurement of breastfeeding** |
| --- | --- | --- | --- |
| PMA-Cohort1-Ethiopia | Ethiopia | This was a prospective population-based cohort study conducted in six Ethiopian regions (Tigray, Oromia, Amhara, SNNP [Southern Nations, Nationalities and Peoples], Afar, and Addis Ababa) between 2019 and 2021 as part of the Performance Monitoring for Action Ethiopia (PMA-Ethiopia) survey. A multistage sampling method was used to sample 2869 (response rate 99.6%) women between the ages of 15 and 49 who were pregnant or had given birth within the previous six months from 32614 households. The pregnant women were followed up at six weeks, six months, and one year postpartum. For recently postpartum women (<6 weeks), the baseline survey and six-week postpartum interview were completed at enrolment. Singleton births were included in this analysis. | Information on infant feeding practices was collected through interviews with mothers at six weeks, six months, and one year postpartum. Among the questions were whether the mother had ever breastfed the child, when they first breastfed after delivery, if they were currently breastfeeding, and what they had fed the child in the previous 24 hours. At the 6-month visit, mothers were asked the age at which they started giving their babies other foods and drinks. At the one-year visit, mothers were asked if they had breastfed their child in the previous 24 hours. |
| PMA-MNH-Ethiopia | Ethiopia | This was a prospective population-based cohort study conducted in the Southern Nations, Nationalities and Peoples' region of Ethiopia between 2016 and 2017 (31). A multistage sampling procedure was used to sample 329 pregnant women from 10 399 households. The study participants were 15-49 years old, at least six months pregnant, and regular members of selected households. Data on pregnancy and sociodemographic characteristics were collected before birth. After delivery, participants were followed up at seven days, six weeks, and six months postpartum, and information about childbirth services, vaccinations, postnatal care, and feeding practices was collected. Singleton births were included in this analysis. | Information on infant feeding practices was collected during the postpartum follow-up visits at seven days, six weeks, and six months after birth. Mothers were asked how long after birth they started breastfeeding and what they fed their child in the 24 hours preceding each visit. Sufficient information was not collected to determine exclusive breastfeeding at six months. |
| Karonga-HDSS-Malawi | Malawi | The Karonga Health and Demographic Surveillance System (HDSS) site is located in northern Malawi(32) and includes 42,000 individuals living in 7000 households(33,34). The population is mainly rural, with residents engaged in subsistence farming, fishing, and trading. The site began with a baseline census between 2002 and 2004, and births to women in the baseline survey were recorded, and the newborns were enrolled. The site conducts ongoing surveillance, gathering data on socioeconomic factors, demographics, household characteristics, and vital events. Data on the children born between 2002 and 2004 were analysed. | Breastfeeding information was collected when the children in the cohort were 0-3 months and again when they were about one year. Mothers were asked if they had ever breastfed the child and the age of the child when other kinds of milk, foods, and drinks were introduced. At the one-year visit, mothers were asked if they breastfed the child in the 24 hours preceding the visit. |
| GPC-Uganda | Uganda | The General Population Cohort (GPC) is a prospective population-based cohort study in rural Southwestern Uganda established in 1989(25). The study site is in Kalungu district, approximately 120 km west of Kampala. The primary economic activities of the residents are subsistence agriculture and small-scale trading. Annual surveys collect routine data on household members and housing characteristics and possessions. In 1999, child health surveys were introduced as part of the GPC to gather detailed information on children under thirteen(35). Data on the children born between 2000 and 2011 were analysed. | Breastfeeding data were collected annually from mothers whose children participated in child surveys. The mothers were asked if they had ever breastfed the child if they were currently breastfeeding, and the age of the child when they stopped breastfeeding. Mothers were also asked the age of the child when other kinds of milk, foods, and liquids were introduced. |
| CIGNIS-Zambia | Zambia | This was a randomised, double-blind, controlled trial conducted in Chilenje, Lusaka, Zambia, between 2005 and 2009(26). It aimed to investigate the effects of locally produced complementary foods on stunting in infants at 18 months of age. Mother-infant pairs were recruited at the Chilenje government clinic if the child was six months old and did not have a severe clinical condition, and the mother consented to feed the child the complementary food for a year and make monthly clinic visits. Overall, 811 mother-infant pairs were recruited and monitored monthly for 12 months. Infants were randomly assigned to receive either a richly fortified locally produced porridge or porridge with conventional fortification. On assessment at age 18 months, there was no difference in the proportion stunted between the intervention and the control groups. Children from both arms of the trial were included in this analysis. | During each monthly clinic visit, mothers were asked about their current breastfeeding status. For mothers who had stopped breastfeeding, additional information was gathered on when they stopped. Data were not collected on when other foods and fluids were introduced. |
| BFPH-Zambia | Zambia | This was a prospective cohort study conducted between 2001 and 2003 in Chilenje, Lusaka, Zambia to investigate the risk for subclinical mastitis, breast milk HIV viral load and postpartum morbidity among HIV-infected and uninfected Zambian women (27,28). Women were recruited from the Chilenje clinic at 32 to 34 weeks gestation if they lived within the Chilenje clinic area, attended antenatal care at the clinic, knew their HIV status, and planned to breastfeed after delivery. Eleven postpartum follow-up visits (3, 7, 10, and 14 days and at 3, 4, 5, 6, 9, 12, and 16 weeks) were made to monitor and collect maternal and child health information from eligible women up until 16 weeks postpartum. | At each postpartum visit, mothers were asked about infant feeding practices, including whether the child was breastfeeding, and if water, other kinds of milk, foods, or liquids were introduced. The breastfeeding information was collected for four months postpartum. |
