# Supplementary material for: Socioeconomic pattern of breastfeeding in sub-Saharan Africa: an individual participant data meta-analysis of six longitudinal cohorts
Source: BMJ Public Health. 2025 Mar 18;3(1):e001298. doi: 10.1136/bmjph-2024-001298 (PMC12107469; doi:10.1136/bmjph-2024-001298)
Supplement: online supplemental file 3 [file bmjph-3-1-s003.docx]

**Supplementary Table 4: Meta-regression of exclusive breastfeeding ≥4 months**

|  | **Crude OR (95% CI)** | **Adjusted OR (95% CI)** |
| --- | --- | --- |
|  |  |  |
| Breastfeeding outcome: *Exclusive breastfeeding ≥4 months* |  |  |
| Socioeconomic status indicators: *Education* |  |  |
| **Study variables:** |  |  |
| **Birth year of study participants^a^** |  |  |
| Before 2019 | 1.00 | 1.00 |
| 2019 | 0.87 (0.75 - 1.01) | 0.85 (0.69 - 1.04) |
| **Year of study data collection^b^** |  |  |
| Before 2019 | 1.00 | 1.00 |
| 2019 - 2021 | 0.87 (0.75 - 1.01) | 0.86 (0.73 - 1.01)^¥^ |
| **Study extent^a^** |  |  |
| Hospital-based | 1.00 | 1.00 |
| Population-based | 1.09 (0.75 - 1.57) | 1.13 (0.75 - 1.71) |
| **Sample analysed^a^** |  |  |
| <1500 | 1.00 | 1.00 |
| >1500 | 0.99 (0.79 - 1.26) | 1.04 (0.80 - 1.36) |
| **Frequency of breastfeeding measurement^c^** |  |  |
| Monthly | 1.00 | 1.00 |
| Six-monthly | 0.99 (0.70 - 1.43) | 0.96 (0.60 - 1.52) |
| Annually | 1.16 (0.81 - 1.66) | 1.13 (0.75 - 1.71) |
|  |  |  |
| Breastfeeding outcome: *Exclusive breastfeeding ≥4 months* |  |  |
| Socioeconomic status indicators: *Wealth* |  |  |
| **Study variables:** |  |  |
| **Birth year of study participants^a^** |  |  |
| Before 2019 | 1.00 | 1.00 |
| 2019 | 0.99 (0.95 - 1.04) | 1.02 (0.97 - 1.07) |
| **Year of study data collection^b^** |  |  |
| Before 2019 | 1.00 | 1.00 |
| 2019 - 2021 | 0.99 (0.95 - 1.04) | 0.98 (0.92 - 1.05) |
| **Study extent^a^** |  |  |
| Hospital-based | 1.00 | 1.00 |
| Population-based | 1.15 (0.87 - 1.51) | 1.27 (0.95 - 1.68) |
| **Sample analysed^a^** |  |  |
| <1500 | 1.00 | 1.00 |
| >1500 | 0.91 (0.85 - 0.99) | 0.89 (0.81 - 0.97)^§^ |
| **Frequency of breastfeeding measurement^c^** |  |  |
| Monthly | 1.00 | 1.00 |
| Six-monthly | 1.15 (0.86 - 1.52) | 1.29 (0.97 - 1.72) |
| Annually | 1.16 (0.88 - 1.55) | 1.27 (0.95 - 1.68) |

^a^Adjusted for sample size, year born, and study design.

^b^Adjusted for study design

^c^Adjusted for sample size

^¥^R-squared=39.2%

^§^R-squared=25.9%
