# Supplementary material for: Socioeconomic pattern of breastfeeding in sub-Saharan Africa: an individual participant data meta-analysis of six longitudinal cohorts
Source: BMJ Public Health. 2025 Mar 18;3(1):e001298. doi: 10.1136/bmjph-2024-001298 (PMC12107469; doi:10.1136/bmjph-2024-001298)
Supplement: online supplemental file 4 [file bmjph-3-1-s004.docx]

**Supplementary Table 3: Cohort-specific risk ratios (RR) from univariable and multivariable Poisson regression models for the association of maternal education and household wealth with breastfeeding indicators.**

|  | **Ever breastfed** | | **Breastfeeding initiation  within 1 hour after birth** | | **Exclusive breastfeeding**  **for ≥4 months** | | **Exclusive breastfeeding**  **for ≥6 months** | | **Continued breastfeeding**  **for ≥1 year** | |
| --- | --- | --- | --- | --- | --- | --- | --- | --- | --- | --- |
|  | **Crude RR**  **(95%CI)** | **Adjusted RR (95%CI)** | **Crude RR**  **(95%CI)** | **Adjusted RR (95%CI)** | **Crude RR**  **(95%CI)** | **Adjusted RR**  **(95%CI)** | **Crude RR**  **(95%CI)** | **Adjusted RR**  **(95%CI)** | **Crude RR**  **(95%CI)** | **Adjusted RR**  **(95%CI)** |
| **PMA- Cohort1-Ethiopia** | |  |  |  |  |  |  |  |  |  |
| **Maternal education** | n=1965 | | n=1990 | | n=1751 | | n=1751 | | n=1945 | |
| None/primary | 1.00 | 1.00 | 1.00 | 1.00 | 1.00 | 1.00 | 1.00 | 1.00 | 1.00 | 1.00 |
| Secondary | 1.01 (0.99 - 1.02) | 1.00 (0.99 - 1.02) | 1.10 (1.00 - 1.21) | 1.11 (1.02 - 1.22) | 1.01 (0.96 - 1.05) | 0.99 (0.95 - 1.04) | 1.00 (0.88 - 1.14) | 1.02 (0.89 - 1.16) | 0.97 (0.95 - 0.99) | 0.99 (0.97 - 1.02) |
| Tertiary | 1.01 (0.99 - 1.03) | 1.00 (0.98 - 1.02) | 0.95 (0.78 - 1.16) | 0.96 (0.78 - 1.17) | 1.04 (1.01 - 1.08) | 1.02 (1.00 - 1.05) | 0.89 (0.73 - 1.09) | 0.94 (0.77 - 1.15) | 0.91 (0.83 - 1.00) | 0.94 (0.87 - 1.03) |
| **Household wealth level** | n=1965 | | n=1990 | | n=1751 | | n=1751 | | n=1945 | |
| Low | 1.00 | 1.00 | 1.00 | 1.00 | 1.00 | 1.00 | 1.00 | 1.00 | 1.00 | 1.00 |
| Middle | 1.01 (0.99 - 1.03) | 1.01 (0.99 - 1.03) | 1.08 (0.96 - 1.22) | 1.09 (0.97 - 1.23) | 1.00 (0.96 - 1.04) | 1.00 (0.96 - 1.04) | 0.99 (0.89 - 1.11) | 1.00 (0.90 - 1.11) | 1.00 (0.98 - 1.01) | 1.00 (0.98 - 1.02) |
| High | 1.02 (1.01 - 1.04) | 1.03 (1.01 - 1.04) | 1.08 (0.96 - 1.21) | 1.08 (0.94 - 1.23) | 1.02 (0.98 - 1.05) | 1.00 (0.97 - 1.04) | 0.97 (0.87 - 1.09) | 1.01 (0.87 - 1.17) | 0.97 (0.95 - 0.99) | 1.00 (0.98 - 1.01) |
| **PMA-MNH-Ethiopia** | |  |  |  |  |  |  |  |  |  |
| **Maternal education** |  |  | n=312 | |  |  |  |  |  |  |
| None/primary | - | - | 1.00 | 1.00 | - | - | - | - | - | - |
| Secondary | - | - | 1.03 (0.73 - 1.45) | 0.98 (0.65 - 1.50) | - | - | - | - | - | - |
| Tertiary | - | - | 1.17 (0.81 - 1.69) | 1.07 (0.74 - 1.53) | - | - | - | - | - | - |
| **Household wealth level** |  |  | n=312 | |  |  |  |  |  |  |
| Low | - | - | 1.00 | 1.00 | - | - | - | - | - | - |
| Middle | - | - | 1.23 (0.93 - 1.63) | 1.25 (0.97 - 1.61) | - | - | - | - | - | - |
| High | - | - | 1.07 (0.77 - 1.49) | 1.05 (0.67 - 1.63) | - | - | - | - | - | - |
| **Karonga - HDSS - Malawi** | |  |  |  |  |  |  |  |  |  |
| **Maternal education** |  | |  |  | n=996 | | n=996 | |  | |
| None/primary | - | - | - | - | 1.00 | 1.00 | 1.00 | 1.00 | - | - |
| Secondary | - | - | - | - | 1.10 (1.00 - 1.21) | 1.14 (1.03 - 1.26) | 1.09 (0.90 - 1.32) | 1.15 (0.94 - 1.41) | - | - |
| **Household wealth** |  |  |  |  | n=972 | | n=972 | |  |  |
| Poorest | - | - | - | - | 1.00 | 1.00 | 1.00 | 1.00 | - | - |
| Middle | - | - | - | - | 1.08 (0.97 - 1.20) | 1.07 (0.96 - 1.20) | 1.14 (0.94 - 1.39) | 1.13 (0.92 - 1.38) | - | - |
| Least poor | - | - | - | - | 1.17 (1.06 - 1.30) | 1.15 (1.03 - 1.28) | 1.20 (0.98 - 1.46) | 1.14 (0.92 - 1.40) | - | - |
|  | **Ever breastfed** | | **Breastfeeding initiation  within 1 hour after birth** | | **Exclusive breastfeeding for ≥4 months** | | **Exclusive breastfeeding for ≥6 months** | | **Continued breastfeeding for ≥1 year** | |
|  | **Crude RR**  **(95%CI)** | **Adjusted RR (95%CI)** | **Crude RR**  **(95%CI)** | **Adjusted RR (95%CI)** | **Crude RR**  **(95%CI)** | **Adjusted RR (95%CI)** | **Crude RR**  **(95%CI)** | **Adjusted RR (95%CI)** | **Crude RR**  **(95%CI)** | **Adjusted RR**  **(95%CI)** |
| **GPC-Uganda** | |  |  |  |  |  |  |  |  |  |
| **Maternal education** |  |  |  |  | n=1553 | | n=1553 | | n=1227 | |
| None/primary | - | - | - | - | 1.00 | 1.00 | 1.00 | 1.00 | 1.00 | 1.00 |
| Secondary | - | - | - | - | 1.07 (1.00 - 1.15) | 1.06 (0.99 - 1.14) | 1.10 (0.99 - 1.22) | 1.09 (0.98 - 1.21) | 0.98 (0.93 - 1.04) | 0.99 (0.93 - 1.04) |
| Tertiary | - | - | - | - | 1.32 (1.21 - 1.43) | 1.33 (1.22 - 1.46) | 1.59 (1.40 - 1.80) | 1.61 (1.40 - 1.84) | 0.91 (0.78 - 1.06) | 0.90 (0.78 - 1.05) |
| **Household wealth level** |  |  |  |  | n=3015 | | n=3015 | | n=2318 | |
| Low | - | - | - | - | 1.00 | 1.00 | 1.00 | 1.00 | 1.00 | 1.00 |
| Middle | - | - | - | - | 0.98 (0.92 - 1.04) | 0.98 (0.92 - 1.04) | 0.97 (0.89 - 1.06) | 0.97 (0.89 - 1.06) | 1.00 (0.95 - 1.05) | 0.99 (0.94 - 1.04) |
| High | - | - | - | - | 0.98 (0.94 - 1.03) | 0.99 (0.94 - 1.04) | 0.96 (0.89 - 1.03) | 0.96 (0.89 - 1.03) | 0.98 (0.94 - 1.02) | 0.97 (0.93 - 1.01) |
| **CIGNIS - Zambia** | |  |  |  |  |  |  |  |  |  |
| **Maternal education** | n=811 | |  |  |  |  |  |  | n=760 | |
| None/primary | 1.00 | 1.00 | - | - | - | - | - | - | 1.00 | 1.00 |
| Secondary | 1.00 (0.97 - 1.05) | 0.99 (0.95 - 1.03) | - | - | - | - | - | - | 1.11 (0.98 - 1.26) | 0.98 (0.89 - 1.08) |
| Tertiary | 0.98 (0.94 - 1.03) | 0.96 (0.91 - 1.00) |  |  |  |  |  |  | 1.11 (0.97 - 1.27) | 0.93 (0.84 - 1.03) |
| **Household wealth level** | n=811 | | - | - | - | - | - | - | n=760 | |
| Low | 1.00 | 1.00 |  |  |  |  |  |  | 1.00 | 1.00 |
| Middle | 0.97 (0.92 - 1.02) | 0.95 (0.91 - 1.00) | - | - | - | - | - | - | 0.99 (0.85 - 1.14) | 0.95 (0.84 - 1.06) |
| High | 0.98 (0.94 - 1.02) | 0.96 (0.93 - 1.00) | - | - | - | - | - | - | 1.03 (0.92 - 1.16) | 0.89 (0.82 - 0.97) |
| **BFPH - Zambia** | |  |  |  |  |  |  |  |  |  |
| **Maternal education** |  |  |  |  | n=373 | |  |  |  |  |
| None/primary | - | - | - | - | 1.00 | 1.00 | - | - | - | - |
| Secondary | - | - | - | - | 1.18 (0.77 - 1.79) | 1.13 (0.74 - 1.72) | - | - | - | - |
| Tertiary |  |  |  |  | 0.96 (0.59 - 1.54) | 0.87 (0.54 - 1.42) | - | - | - | - |
| **Household wealth level** | - | - | - | - | n=373 | |  |  |  |  |
| Low |  |  |  |  | 1.00 | 1.00 | - | - | - | - |
| Middle | - | - | - | - | 0.91 (0.69 - 1.21) | 0.91 (0.69 - 1.22) | - | - | - | - |
| High | - | - | - | - | 0.61 (0.25 - 1.48) | 0.60 (0.24 - 1.45) | - | - | - | - |

PMA-Cohort1-Ethiopia: adjusted for child sex, place of residence, maternal age, parity, and maternal marital status.

PMA-MNH-Ethiopia: adjusted for child sex, place of residence, maternal age, parity, and maternal marital status.

Karonga-HDSS-Malawi: adjusted for child sex, birth order, distance to a tarmac road, maternal HIV status, and maternal age.

GPC-Uganda: adjusted for child sex, maternal age, maternal HIV status, and maternal marital status.

BFPH-Zambia: adjusted for child sex, maternal age, maternal HIV status, and maternal marital status.

CIGNIS-Zambia: adjusted for child sex, child HIV status, maternal HIV status, number of siblings, Maternal age, and maternal marital status.
