# Supplementary material for: Socioeconomic pattern of breastfeeding in sub-Saharan Africa: an individual participant data meta-analysis of six longitudinal cohorts
Source: BMJ Public Health. 2025 Mar 18;3(1):e001298. doi: 10.1136/bmjph-2024-001298 (PMC12107469; doi:10.1136/bmjph-2024-001298)
Supplement: online supplemental file 5 [file bmjph-3-1-s005.docx]

**Supplementary Table 2 Distribution of breastfeeding indicators across participants characteristics by cohorts**

|  | **Total** | **Ever breastfed** | **Breastfeeding initiation within 1 hour after birth** | **Exclusive breastfeeding for ≥4 months** | **Exclusive breastfeeding for ≥6 months** | **Continued breastfeeding for ≥1 year** |
| --- | --- | --- | --- | --- | --- | --- |
| **PMA-Cohort1-Ethiopia*** |  |  |  |  |  |  |
| **Child sex** |  |  |  |  |  |  |
| Male | 1049 (51.1) | 1004 (97.9) | 695 (67.1) | 863 (94.3) | 617 (67.4) | 974 (97.0) |
| Female | 1004 (48.9) | 961 (98.5) | 670 (67.7) | 813 (93.9) | 594 (68.6) | 953 (98.0) |
| **Residence** |  |  |  |  |  |  |
| Urban | 465 (22.2) | 442 (99.2) | 308 (70.0) | 394 (95.2) | 269 (65.0) | 424 (92.7) |
| Rural | 1631 (77.8) | 1560 (98.0) | 1056 (66.7) | 1317 (93.9) | 965 (68.7) | 1544 (98.8) |
| **Maternal age** |  |  |  |  |  |  |
| <20 | 192 (9.3) | 176 (98.4) | 122 (63.6) | 156 (95.6) | 113 (69.0) | 180 (98.0) |
| 20-29 | 1094 (53.3) | 1062 (98.8) | 752 (69.6) | 910 (94.9) | 662 (69.1) | 1030 (96.8) |
| ≥30 | 767 (37.4) | 728 (97.2) | 491 (65.2) | 611 (92.6) | 435 (66.0) | 719 (98.4) |
| **Number of previous births** |  |  |  |  |  |  |
| 0 | 358 (17.1) | 336 (97.9) | 214 (64.4) | 302 (95.2) | 201 (63.3) | 323 (93.9) |
| 1 | 458 (21.9) | 443 (99.6) | 291 (65.0) | 383 (96.7) | 276 (69.7) | 439 (97.8) |
| 2-3 | 588 (28.0) | 560 (97.9) | 401 (70.2) | 482 (94.8) | 362 (71.3) | 555 (97.8) |
| ≥4 | 691 (33.0) | 663 (97.8) | 457 (68.1) | 545 (91.4) | 395 (66.2) | 651 (98.7) |
| **Marital status** |  |  |  |  |  |  |
| Married | 1993 (95.2) | 1902 (98.1) | 1309 (67.8) | 1626 (94.1) | 1163 (67.3) | 1876 (97.6) |
| Unmarried | 100 (4.8) | 98 (100.0) | 55 (59.3) | 84 (94.6) | 71 (79.2) | 91 (94.3) |
| **PMA-MNH-Ethiopia*** |  |  |  |  |  |  |
| **Child sex** |  |  |  |  |  |  |
| Male | 187 (58.1) | - | 111 (61.4) | - | - | - |
| Female | 135 (41.9) | - | 88 (65.4) | - | - | - |
| **Residence** |  |  |  |  |  |  |
| Urban | 37 (11.3) | - | 25 (72.1) | - | - | - |
| Rural | 292 (88.7) | - | 174 (62.0) | - | - | - |
| **Maternal age** |  |  |  |  |  |  |
| <20 | 37 (11.2) | - | 22 (66.0) | - | - | - |
| 20-29 | 169 (51.5) | - | 112 (69.1) | - | - | - |
| ≥30 | 123 (37.3) | - | 65 (54.2) | - | - | - |
| **Number of previous births** |  |  |  |  |  |  |
| 0 | 0 (0.0) | - | 0 (0.0) | - | - | - |
| 1 | 67 (20.6) | - | 44 (68.4) | - | - | - |
| 2-3 | 82 (25.4) | - | 48 (60.8) | - | - | - |
| ≥4 | 175 (54.0) | - | 106 (62.2) | - | - | - |
| **Marital status** |  |  |  |  |  |  |
| Married | 319 (97.1) | - | 190 (62.0) | - | - | - |
| Unmarried | 10 (2.9) | - | 9 (96.7) | - | - | - |
| **Karonga HDSS-Malawi** |  |  |  |  |  |  |
| **Child sex** |  |  |  |  |  |  |
| Female | 670 (47.9) | 667 (99.7) | - | 477 (71.5) | 271 (40.6) | 646 (99.7) |
| Male | 730 (52.1) | 725 (99.4) | - | 498 (68.7) | 282 (38.9) | 697 (99.0) |
| **Birth order** |  |  |  |  |  |  |
| 1 | 325 (24.6) | 324 (100.0) | - | 207 (63.9) | 113 (34.9) | 314 (98.4) |
| 2-3 | 444 (33.6) | 441 (99.3) | - | 313 (71.0) | 179 (40.6) | 423 (99.8) |
| 4 or more | 551 (41.7) | 547 (99.5) | - | 395 (72.2) | 226 (41.3) | 529 (99.6) |
| **Distance to Tarmac road** |  |  |  |  |  |  |
| Within 1km | 714 (51.0) | 709 (99.6) | - | 520 (73.3) | 312 (44.0) | 680 (99.3) |
| More than 1km | 685 (49.0) | 682 (99.6) | - | 454 (66.6) | 240 (35.2) | 662 (99.4) |
| **Maternal HIV status** |  |  |  |  |  |  |
| Negative | 1138 (81.3) | 1133 (99.6) | - | 775 (68.4) | 435 (38.4) | 1095 (99.5) |
| Positive | 44 (3.2) | 44 (100.0) | - | 33 (75.0) | 22 (50.0) | 43 (100.0) |
| Unknown | 217 (15.5) | 214 (99.1) | - | 166 (77.6) | 95 (44.4) | 204 (98.1) |
| **Maternal age** |  |  |  |  |  |  |
| <20 | 213 (20.4) | 212 (100.0) | - | 135 (63.7) | 76 (35.8) | 208 (100.0) |
| 20-29 | 580 (55.6) | 577 (99.6) | - | 399 (69.1) | 214 (37.1) | 555 (99.8) |
| ≥30 | 251 (24.0) | 249 (99.2) | - | 181 (72.7) | 106 (42.6) | 242 (100.0) |
|  |  |  |  |  |  |  |
|  | **Total** | **Ever breastfed** | **Breastfeeding initiation within 1 hour after birth** | **Exclusive breastfeeding for ≥4 months** | **Exclusive breastfeeding for ≥6 months** | **Continued breastfeeding for ≥1 year** |
| **GPC-Uganda** |  |  |  |  |  |  |
| **Child sex** |  |  |  |  |  |  |
| Male | 4798 (50.8) | 3523 (99.3) | - | 2148 (71.4) | 1670 (55.5) | 1622 (68.4) |
| Female | 4639 (49.2) | 3288 (99.3) | - | 2056 (73.0) | 1582 (56.2) | 1430 (68.1) |
| **Maternal age** |  |  |  |  |  |  |
| <20 | 658 (16.7) | 579 (99.8) | - | 378 (75.9) | 267 (53.6) | 328 (81.8) |
| 20-29 | 2173 (55.2) | 1923 (100.0) | - | 1301 (74.8) | 1030 (59.2) | 1116 (84.0) |
| ≥30 | 1108 (28.1) | 1007 (99.9) | - | 629 (66.2) | 478 (50.3) | 573 (84.0) |
| **Marital status** |  |  |  |  |  |  |
| Married | 3008 (77.6) | 2715 (100.0) | - | 1816 (72.7) | 1407 (56.3) | 1608 (84.3) |
| Unmarried | 869 (22.4) | 757 (99.9) | - | 472 (71.6) | 353 (53.6) | 399 (81.8) |
| **Maternal HIV status** |  |  |  |  |  |  |
| Positive | 267 (2.8) | 211 (100.0) | - | 133 (68.9) | 91 (47.1) | 95 (62.5) |
| Negative | 3672 (38.9) | 3294 (99.9) | - | 2173 (72.7) | 1682 (56.2) | 1919 (85.0) |
| Unknown | 5498 (58.3) | 3306 (98.6) | - | 1898 (71.9) | 1479 (56.0) | 1038 (50.4) |
| **CIGNIS-Zambia** |  |  |  |  |  |  |
| **Child sex** |  |  |  |  |  |  |
| Male | 385 (47.5) | 364 (94.5) | - | - | - | 235 (64.6) |
| Female | 426 (52.5) | 396 (93.0) | - | - | - | 262 (66.2) |
| **Child HIV status at 18 months** |  |  |  |  |  |  |
| Negative | 599 (73.9) | 561 (93.7) | - | - | - | 437 (77.9) |
| Positive | 18 (2.2) | 16 (88.9) | - | - | - | 8 (50.0) |
| Unknown | 194 (23.9) | 183 (94.3) | - | - | - | 52 (28.4) |
| **Number of siblings** |  |  |  |  |  |  |
| 0 | 676 (83.4) | 633 (93.6) | - | - | - | 412 (65.1) |
| 1-2 | 133 (16.4) | 125 (94.0) | - | - | - | 83 (66.4) |
| 3 | 2 (0.2) | 2 (100.0) | - | - | - | 2 (100.0) |
| **Maternal age** |  |  |  |  |  |  |
| <20 | 90 (11.1) | 88 (97.8) | - | - | - | 53 (60.2) |
| 20-29 | 504 (62.1) | 481 (95.4) | - | - | - | 320 (66.5) |
| ≥30 | 217 (26.8) | 191 (88.0) | - | - | - | 124 (64.9) |
| **Maternal HIV status** |  |  |  |  |  |  |
| Negative | 564 (69.5) | 561 (99.5) | - | - | - | 419 (74.7) |
| Positive | 177 (21.8) | 131 (74.0) | - | - | - | 29 (22.1) |
| Unknown | 70 (8.6) | 68 (97.1) | - | - | - | 49 (72.1) |
| **Marital status** |  |  |  |  |  |  |
| Married | 204 (25.2) | 188 (92.2) | - | - | - | 108 (57.5) |
| Unmarried | 607 (74.8) | 572 (94.2) | - | - | - | 389 (68.0) |
| **BFPH-Zambia** |  |  |  |  |  |  |
| **Child sex** |  |  |  |  |  |  |
| Female | 197 (52.8) | - | - | 74 (37.6) | - | - |
| Male | 176 (47.2) | - | - | 54 (30.7) | - | - |
| **Maternal age** |  |  |  |  |  |  |
| <20 | 69 (18.4) | - | - | 23 (33.3) | - | - |
| 20-29 | 253 (67.7) | - | - | 89 (35.2) | - | - |
| ≥30 | 52 (13.9) | - | - | 16 (30.8) | - | - |
| **Maternal HIV status** |  |  |  |  |  |  |
| Negative | 188 (50.3) | - | - | 66 (35.1) | - | - |
| Positive | 186 (49.7) | - | - | 62 (33.3) | - | - |
| **Marital status** |  |  |  |  |  |  |
| Married | 273 (73.0) | - | - | 89 (32.6) | - | - |
| Unmarried | 101 (27.0) | - | - | 39 (38.6) | - | - |

Not all participants in the total sample had information on all the breastfeeding indicators.

*Percentages and counts are weighted estimates
